# Association between excessive fetal growth and maternal cancer in Shanghai, China: a large, population-based cohort study

**DOI:** 10.1038/s41598-023-33664-4

**Published:** 2023-05-13

**Authors:** Naisi Qian, Qing Yang, Lei Chen, Shan Jin, Jiaying Qiao, Renzhi Cai, Chunxiao Wu, Huiting Yu, Kai Gu, Chunfang Wang

**Affiliations:** 1 Department of Vital Statistics, Institute of Health Information, Shanghai Municipal Center for Disease Control and Prevention, West Zhongshan Rd. No 1380, Changning District, Shanghai, China; 2 Department of Oncology, Institute of Chronic Disease Control and Prevention, Shanghai Municipal Center for Disease Control and Prevention, West Zhongshan Rd. No 1380, Changning District, Shanghai, China

**Keywords:** Cancer epidemiology, Risk factors

## Abstract

The prevalence of high birth weight or large for gestational age (LGA) infants is increasing, with increasing evidence of pregnancy-related factors that may have long-term impacts on the health of the mother and baby. We aimed to determine the association between excessive fetal growth, specifically LGA and macrosomia, and subsequent maternal cancer by performing a prospective population-based cohort study. The data set was based on the Shanghai Birth Registry and Shanghai Cancer Registry, with medical records from the Shanghai Health Information Network as a supplement. Macrosomia and LGA prevalence was higher in women who developed cancer than in women who did not. Having an LGA child in the first delivery was associated with a subsequently increased risk of maternal cancer (hazard ratio [HR] = 1.08, 95% confidence interval [CI] 1.04–1.11). Additionally, in the last and heaviest deliveries, there were similar associations between LGA births and maternal cancer rates (HR = 1.08, 95% CI 1.04–1.12; HR = 1.08, 95% CI 1.05–1.12, respectively). Furthermore, a substantially increased trend in the risk of maternal cancer was associated with birth weights exceeding 2500 g. Our study supports the association between LGA births and increased risks of maternal cancer, but this risk requires further investigation.

## Introduction

During pregnancy, significant anatomical and physiological changes occur in pregnant women to nurture and accommodate the development of the fetus^1,2^. There is increasing evidence that pregnancy-related factors may have a long-term impact on the health of mother and offspring, with multiparity established as a protective factor for breast, ovarian, and endometrial cancer^3^. However, other studies have shown that excessive fetal growth (EFG) is associated with an increased risk of developing hormone-related cancers in mothers, such as breast, thyroid and ovarian cancer^4–6^. Moreover, a large cohort study in Sweden found that EFG was independently associated with a higher risk of colorectal cancer^7^. The potential etiology of the different types of cancer may be due to the different influences of rapidly increasing levels of estrogen, progesterone, insulin-like growth factor-1 (IGF-1), and leptin during pregnancy. However, few studies have evaluated the association between fetal growth and the total risk of all cancers in mothers.

We hypothesized that fetal growth levels may serve as an indicator of estrogen and progesterone levels, and that birth weight and large for gestational age (LGA) weights could be used to indicate fetal growth levels. As the average worldwide birth weight increases^8,9^ and the complex relationship between pregnancy characteristics and different types of cancer grows, it is urgent to investigate the influence of birth weight on the total risk of all cancers. Using a large population-based, data-linked cohort, we aimed to assess the association between the birth weight of infants and the long-term risk of cancers in mothers, ultimately aiming to discover a birth weight that may reduce future maternal cancer risks.

## Methods

### Study design and data collection

We performed a population-based, prospective cohort study to explore the associations between EFG, specifically LGA and macrosomia, during pregnancy and maternal cancer after childbirth. The birth data was set as the basic database, and the mother’s national identification number (ID) was used to link maternal cancer information from the cancer registry information and medical records in the Shanghai Health Information Network (SHIN). This method and its application in similar studies in Shanghai has been commended for its excellent performance in managing and analyzing an extensive database^10,11^.

**Figure 1:**
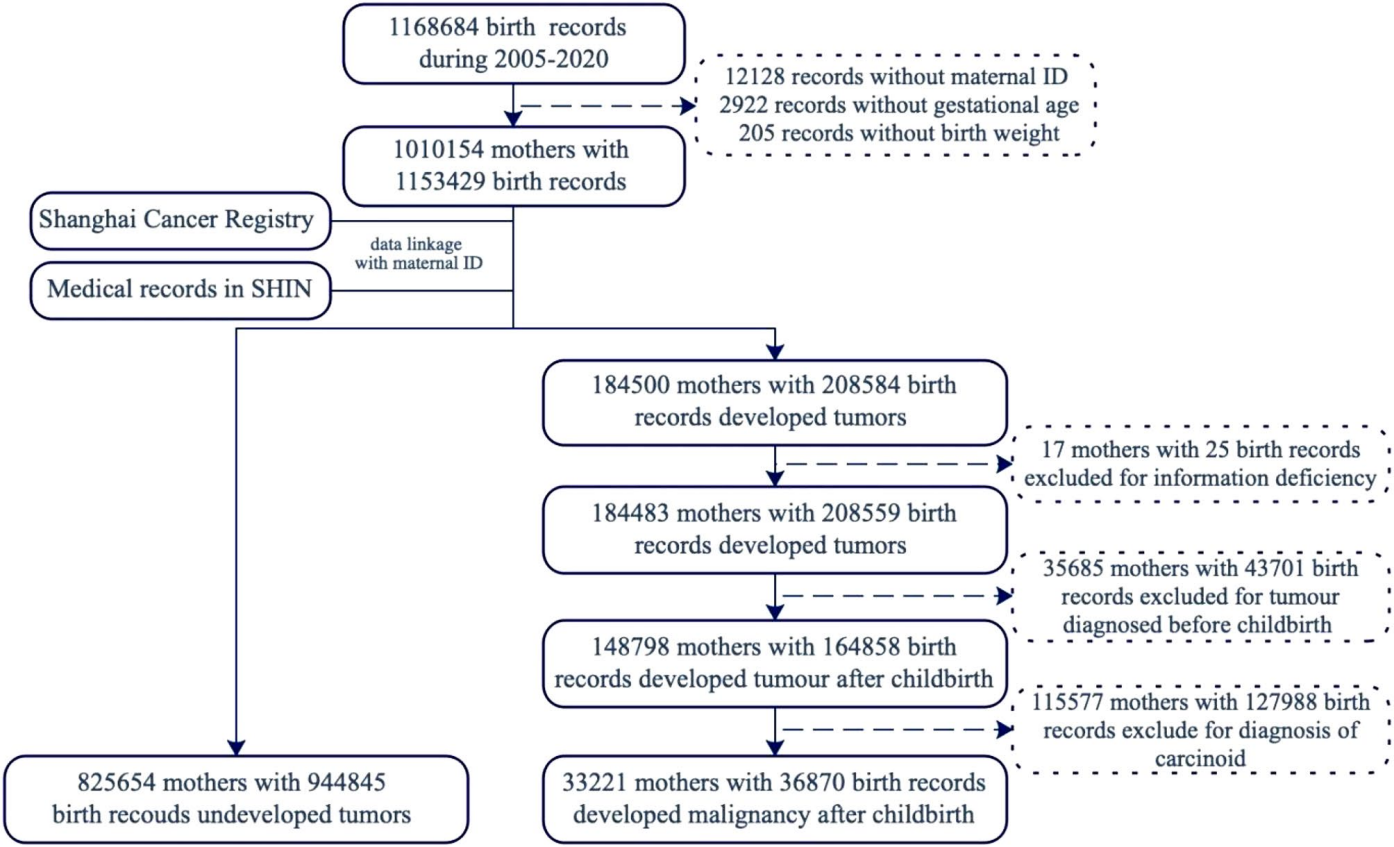
Technical route on inclusion and exclusion of the study object. *The maximum years of follow-up was 15.

Birth data was obtained from the Shanghai Birth Registry (SBR) of the Shanghai Municipal Center for Disease Control and Prevention (SCDC), which is a legislated population-based surveillance system that was established in 2003. The SBR registers all live births with no limitations to birth weight or gestational age at birth. Birth information included date of birth, infant’s sex, weight and length at birth, gestational age at birth, birth defects, embryo number, mode of delivery, gravidity, parity, and socio-demographic characteristics of the infant’s parents. Between January 1, 2005 and December 31, 2020, 1,168,684 live births born to household-registered women in Shanghai were registered in this system (Fig. 1); 15,225 participants were excluded due to incomplete birth information (maternal national ID, gestational age, or birth weight). Ultimately, 1,153,429 live births born to 1,010,154 mothers were included to link with cancer information.

Cancer data were verified from multiple sources according to the registry data from the Shanghai Cancer Registry (SCR) of the SCDC and medical records in SHIN. As a member of the International Association of Cancer Registries (IACR, 150 Cours Albert Thomas 69372 Lyon Cedex 08 France, http://www.iacr.com.fr) and a statutory population-based cancer registry, the SCR covers all registered permanent Shanghai residents^12^. For each patient, the following data were collected: basic demographics, primary site of cancer (coded according to the International Classification of Disease, Revision 10, ICD-10), incidence date, basis of cancer diagnosis, and reporting hospital. More than 75% of cancer diagnoses were verified using pathology, and more than 90% were confirmed as the primary diagnosis.

SHIN was established in 2011 by the Shanghai Municipal Health Committee and covers all public and private hospitals in Shanghai, collecting their outpatient, hospital admission, and discharge records. The medical records in SHIN contain information on patients’ national ID, demographic characteristics, date of visit, physical examinations, imaging results, and diagnosis of each case (coded according to the International Classification of Diseases, 10th edition).

Cancers, identified by registered cancer cases and all diagnoses of malignant cancer (ICD-10 codes: C00-C97) and benign tumors (ICD-10 codes: D00-D48) reported in SHIN for the first time, were linked to 1,153,429 live births born to 1,010,154 mothers by maternal ID. Mothers with a history of tumors before delivery and with a history of benign tumors were excluded. In the end, 33,221 mothers (with 36,870 live births) identified with maternal malignant cancer diagnoses were included, compared with 825,654 mothers (with 944,845 live births) without maternal tumors (Fig. 1).

This study was approved by the Ethics Review Committee of the Shanghai Municipal Centre for Disease Control and Prevention. Following the principles of ethical review in China, it is not necessary to reacquire informed consent from participants for retrospective studies based on population registry information. Therefore, the requirement to obtain written informed consent was waived by the Ethics Review Committee of the Shanghai Municipal Centre for Disease Control and Prevention.

All methods were performed in accordance with the relevant guidelines and regulations.

### Exposure and outcome definition

LGA weights and macrosomia were used as the main indices to identify the diverse impacts of EFG during pregnancy on maternal cancer after delivery. LGA was defined as having a birth weight > the 90th percentile for gestational age based on a sex-specific reference curve for normal fetal growth from a large population-based study in china^13^. Macrosomia was defined as an infant with a birth weight of over 4000 g.

Three data sets were created based on multiple birth records of the mothers: (1) the first delivery, including the first birth records of the mothers; (2) the last delivery, including the last birth records of the mothers; and (3) the heaviest delivery, including the birth records of the heaviest child of the mothers. As more than 85% of the mothers had one child only, the overlap between the first and second groups exceeded 85%.

### Statistical analysis

The birth characteristics among mothers who did and did not develop cancer were described in the first, last, and heaviest deliveries. Chi-square tests were used to compare the differences in birth characteristics, and Fisher’s exact tests were used to assess the significance of the differences when the expected number of any of the cells was below five. However, no analyses on the interactions between birth characteristics were conducted.

Cox proportional hazard models were used to estimate the association between the birth of an LGA or macrosomia infant and the maternal risk of cancer by calculating hazard ratios (HR) and the 95% confidence interval (95% CI), adjusting for potential confounders. The following pregnancy factors were included in the model as covariates (categorical): year of childbirth, maternal age, education level, number of childbirths, history of abortion (including both induced abortion and spontaneous abortion), and number of embryos. Additionally, the time until cancer during follow-up was the dependent variable. To assess heterogeneity effects by maternal age, we used a stratified analysis with each maternal age group. The results were similar to those obtained using an analysis with maternal age serving as a covariate, reported herein. The maternal age and birth weight were set as classification variables in the Cox proportional hazard models for estimating the maternal cancer risk in each group, but were set as continuous variables for estimating the trend.

All tests were two-tailed, and the hypothesis was considered statistically significant if *p* values were < 0.05. Data preparation and analyses were performed using SAS version 9.4.

## Results

This population-based cohort included 858,875 women from 2005 to 2020, of which 33,221 (3.87%) developed cancer. At the end of follow-up, the average age of the women who did not develop cancer was 37.67 years (SD 4.94, range 15.70–68.39), and the average age at cancer diagnosis was 35.82 years (SD 4.99, range 19.51–65.29; Table 1). The average follow-up time from the first delivery was 9.17 years (SD 4.25, range 1.00–17.00) in women without a cancer diagnosis and 7.00 years (SD 3.92, range 0.01–16.56) in women who developed cancer.

**Table 1:** Birth characteristics in the first, last and heaviest delivery, by mother developed and undeveloped cancer.

Variable	Label	The first delivery					The last delivery					The heaviest delivery				
		Women undeveloped cancer		Women developed cancer		*p* value	Women undeveloped cancer		Women developed cancer		*p* value	Women undeveloped cancer		Women developed cancer		*p* value
		N	(%)	N	(%)		N	(%)	N	(%)		N	(%)	N	(%)	
Total		825,654	100	33,221	100		825,654	100	33,221	100		825,654	100	33,221	100	
Year of childbirth	2005–2009	243,064	29.44	14,192	42.72	< 0.0001	207,233	25.10	12,849	38.68	< 0.0001	224,446	27.18	13,490	40.61	< 0.0001
	2010–2014	309,045	37.43	13,223	39.80		281,973	34.15	12,911	38.86		294,783	35.70	13,062	39.32	
	2015–2020	273,545	33.13	5806	17.48		336,448	40.75	7461	22.46		306,425	37.11	6669	20.07	
Mother’s age at birth	Mean/Std	28.50	4.02	28.82	4.07	< 0.0001	29.15	4.19	29.29	4.18	< 0.0001	28.84	4.12	29.06	4.13	< 0.0001
Mother’s education level	Middle school and below	185,452	22.52	7578	22.84	0.3682	181,054	21.99	7490	22.58	0.0280	183,185	22.24	7546	22.75	0.0875
	College and higher	638,140	77.48	25,596	77.16		642,462	78.01	25,683	77.42		640,384	77.76	25,624	77.25	
	Missing	2062		47			2138		48			2085		51		
Number of childbirths	1	708,860	85.85	29,636	89.21	< 0.0001	708,860	85.85	29,636	89.21	< 0.0001	708,860	85.85	29,636	89.21	< 0.0001
	2	114,500	13.87	3521	10.60		114,500	13.87	3521	10.60		114,500	13.87	3521	10.60	
	≥ 3	2294	0.28	64	0.19		2294	0.28	64	0.19		2294	0.28	64	0.19	
History of abortion	None	546,914	66.36	21,201	63.87		546,914	66.36	21,201	63.87		546,914	66.36	21,201	63.87	
	1	191,600	23.25	8113	24.44	< 0.0001	191,600	23.25	8113	24.44	< 0.0001	191,600	23.25	8113	24.44	< 0.0001
	2	62,329	7.56	2824	8.51		62,329	7.56	2824	8.51		62,329	7.56	2824	8.51	
	≥ 3	23,351	2.83	1054	3.18		23,351	2.83	1054	3.18		23,351	2.83	1054	3.18	
	Missing	1460		29			1460		29			1460		29		
Age at last follow-up	Mean/Std	37.67	4.94	35.82	4.99		37.67	4.93	35.81	4.99		37.67	4.93	35.82	4.99	
Year of follow-up	Mean/Std	9.17	4.25	7	3.92		8.51	4.32	6.52	3.94		8.83	4.30	6.75	3.93	
	< 1 year			1544	4.65	< 0.0001			1886	5.68	< 0.0001			1715	5.16	< 0.0001
	1–3 years	71,482	8.66	4678	14.08		91,191	11.04	5689	17.12		81,816	9.91	5214	15.69	
	3–5 years	90,538	10.97	5411	16.29		115,512	13.99	5981	18.00		103,376	12.52	5698	17.15	
	5 years-	663,634	80.38	21,588	64.98		618,951	74.96	19,665	59.19		640,462	77.57	20,594	61.99	
Number of embryos	Single	812,847	98.45	32,715	98.48	0.6733	812,232	98.38	32,696	98.42	0.5578	812,982	98.47	32,720	98.49	0.5647
	Twins	12,708	1.54	501	1.51		13,318	1.61	519	1.56		12,571	1.52	496	1.49	
	Multiple	69	0.01	5	0.02		74	0.01	5	0.02		71	0.01	5	0.02	
	Missing	30					30		1			30				
Child gender	Boy	425,477	51.53	17,123	51.54	0.2596	430,173	52.10	17,319	52.13	0.8765	434,241	52.59	17,445	52.51	0.9375
	Girl	400,170	48.47	16,098	48.46		395,476	47.90	15,902	47.87		391,409	47.41	15,776	47.49	
	Other	7	0.00				5	0.00				4	0.00			
Caesarian	Yes	460,636	55.86	20,401	61.44	< 0.0001	464,266	56.30	20,482	61.68	< 0.0001	462,791	56.12	20,458	61.61	< 0.0001
Gestational age	Mean/Std	38.75	1.46	38.69	1.46		38.69	1.44	38.65	1.44		38.76	1.41	38.7	1.42	
	< 28 weeks	456	0.06	21	0.06	0.0659	378	0.05	15	0.05	0.5349	316	0.04	12	0.04	0.0060
	28–33 weeks	2718	0.33	115	0.35		2685	0.33	120	0.36		2352	0.28	106	0.32	
	34–36 weeks	39,990	4.84	1680	5.06		41,626	5.04	1709	5.14		37,721	4.57	1606	4.83	
	37–41 weeks	780,811	94.57	31,325	94.29		779,493	94.41	31,308	94.24		783,666	94.91	31,419	94.58	
	42-weeks	1679	0.20	80	0.24		1472	0.18	69	0.21		1599	0.19	78	0.23	
Premature birth	Yes	43,164	5.23	1816	5.47	0.0117	44,689	5.41	1844	5.55	0.3344	40,389	4.89	1724	5.19	0.0003
Birth weight	Mean/Std	3318.32	455.58	3334.66	457.61		3321.01	455.20	3337.36	457.11		3345.42	449.48	3356.25	451.07	
	< 1500	2507	0.30	92	0.28	< 0.0001	2375	0.29	82	0.25	< 0.0001	1920	0.23	67	0.20	< 0.0001
	1500–1999	4967	0.60	214	0.64		5014	0.61	222	0.67		4072	0.49	185	0.56	
	2000–2499	20,844	2.52	786	2.37		21,056	2.55	779	2.34		18,063	2.19	692	2.08	
	2500–2999	139,780	16.93	5320	16.01		138,198	16.74	5265	15.85		130,555	15.81	5056	15.22	
	3000–3499	374,250	45.33	15,013	45.19		374,022	45.30	15,007	45.17		369,308	44.73	14,837	44.66	
	3500–3999	232,975	28.22	9600	28.90		234,106	28.35	9645	29.03		246,048	29.80	10,022	30.17	
	4000–4499	45,517	5.51	1975	5.95		45,979	5.57	1992	6.00		50,277	6.09	2125	6.40	
	4500–4999	4814	0.58	221	0.67		4904	0.59	229	0.69		5411	0.66	237	0.71	
Low birth weight	Yes	28,318	3.43	1092	3.29	0.1794	28,445	3.45	1083	3.26	0.0557	24,055	2.91	944	2.84	0.7327
Macrosomia	Yes	45,951	5.57	1989	5.99	0.0004	46,590	5.64	2020	6.08	< 0.0001	50,998	6.18	2147	6.46	< 0.0001
Small than gestational age	Yes	82,386	9.98	2993	9.01	< 0.0001	77,636	9.40	2860	8.61	< 0.0001	72,895	8.83	2709	8.15	< 0.0001
Large than gestational age	Yes	85,816	10.39	3967	11.94	< 0.0001	91,548	11.09	4161	12.53	< 0.0001	95,529	11.57	4285	12.90	< 0.0001

**Figure 2:**
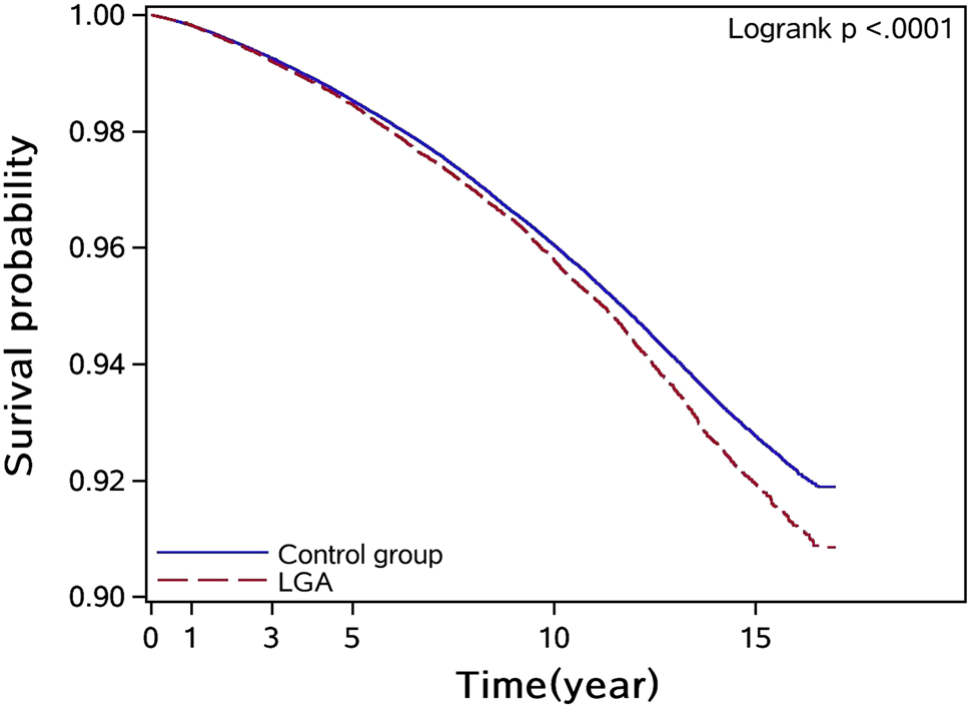
A Kaplan–Meier plot for the incidence of maternal cancer after delivery.

At the first delivery, the average age of women did not develop cancer was 28.5 years, while the women who developed cancer tended to give birth at the relatively late age of 28.82 years. The percentage of women having one child was more in those who developed cancer (89.21%) compared with women who did not develop cancer (85.85%). More often, women who developed cancer had a previous abortion or caesarean section. In the first, last, and heaviest deliveries, the prevalence of macrosomia and LGA infants was higher in women who developed cancer compared with women who did not develop cancer. A Kaplan–Meier plot of the incidence of maternal cancer after delivery was analyzed; it can be seen in Fig. 2 that the two curves do not overlap and that the mothers with an LGA history were prone to develop subsequent cancer.

**Figure 3:**
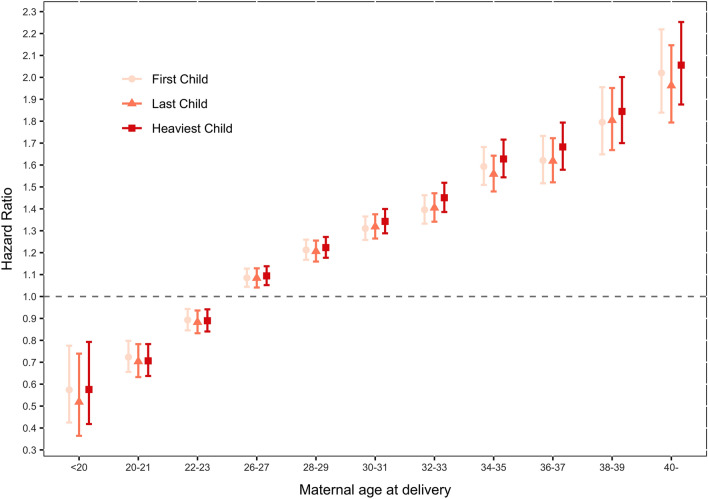
Long-term hazard ratio of cancer after childbirth, according to maternal age at delivery. *Maternal age at delivery between 24 and 25 was control group. *The association between maternal age at delivery and maternal cancer risk is tested to be linear by regression analysis In the First Child group, b = 0.12, adjusted R^2^ = 0.99, t = 30.51, *p* < 0.0001. In the Last Child group, b = 0.12, adjusted R^2^ = 0.99, t = 36.65, *p* < 0.0001. In the Heaviest Child group, b = 0.13, adjusted R^2^ = 0.99, t = 42.33, *p* < 0.0001).

In all three groups, with increasing childbearing age, the risk of cancer after delivery dramatically increased (average of 4.5%, 95% CI 4.2–4.8%, risk increase per 2 years, data not shown; Fig. 3). Compared with delivering between the ages of 24 and 26 years, we found that delivering before 24 years of age decreased long-term cancer risk, while delivering after 26 years increased long-term cancer risk. HRs increased linearly with maternal age at delivery, and the HR increased by 0.12, 0.12 and 0.13 for every 2 year delay in the first, last and heaviest child group, respectively.

The annual cancer incidence rate of mothers who did not give birth to LGA infants in the first delivery was 4.21 (95% CI 4.16–4.25) per 1000 person-years, while those who did give birth to LGA infants in the first delivery had a 4.66 (95% CI 4.52–4.80) per 1000 person-years incidence (Table 2). Having an LGA infant in the first delivery was associated with a subsequent increased risk of maternal cancer (HR = 1.08, 95% CI 1.04–1.11). Regarding the last and heaviest delivery, there was a similar association between the birth of LGA infants and maternal cancer rates (HR = 1.08, 95% CI 1.04–1.12 and HR = 1.08, 95% CI 1.05–1.12, respectively). However, in this study, we did not find an association between the higher risk of subsequent maternal cancer and fetal macrosomia in the first, last, and heaviest delivery.

**Table 2:** Incidence and risk of maternal cancer events after the birth of macrosomia or LGA.

			Number of events	Incidence per 1000 person-years	Crude hazard ratio	Adjusted hazard ratio
First delivery	LGA	Unexposed	29,254	4.21	Ref	Ref
		Exposed	3967	4.66	1.09 (1.06, 1.13)	1.08 (1.04, 1.11)
	Macrosomia	Unexposed	31,232	4.25	Ref	Ref
		Exposed	1989	4.39	1.02 (0.97, 1.07)	1.02 (0.97, 1.07)
Last delivery	LGA	Unexposed	29,060	4.53	Ref	Ref
		Exposed	4161	5.03	1.10 (1.07, 1.14)	1.08 (1.04, 1.11)
	Macrosomia	Unexposed	31,201	4.57	Ref	Ref
		Exposed	2020	4.75	1.03 (0.98, 1.08)	1.03 (0.98, 1.07)
Heaviest delivery	LGA	Unexposed	28,936	4.37	Ref	Ref
		Exposed	4285	4.80	1.09 (1.06, 1.13)	1.08 (1.05, 1.12)
	Macrosomia	Unexposed	31,074	4.42	Ref	Ref
		Exposed	2147	4.49	1.01 (0.97, 1.05)	1.02 (0.98, 1.07)

Adjusted by year of childbirth, maternal age, education level, number of childbirths, history of abortion and number of embryos.

**Figure 4:**
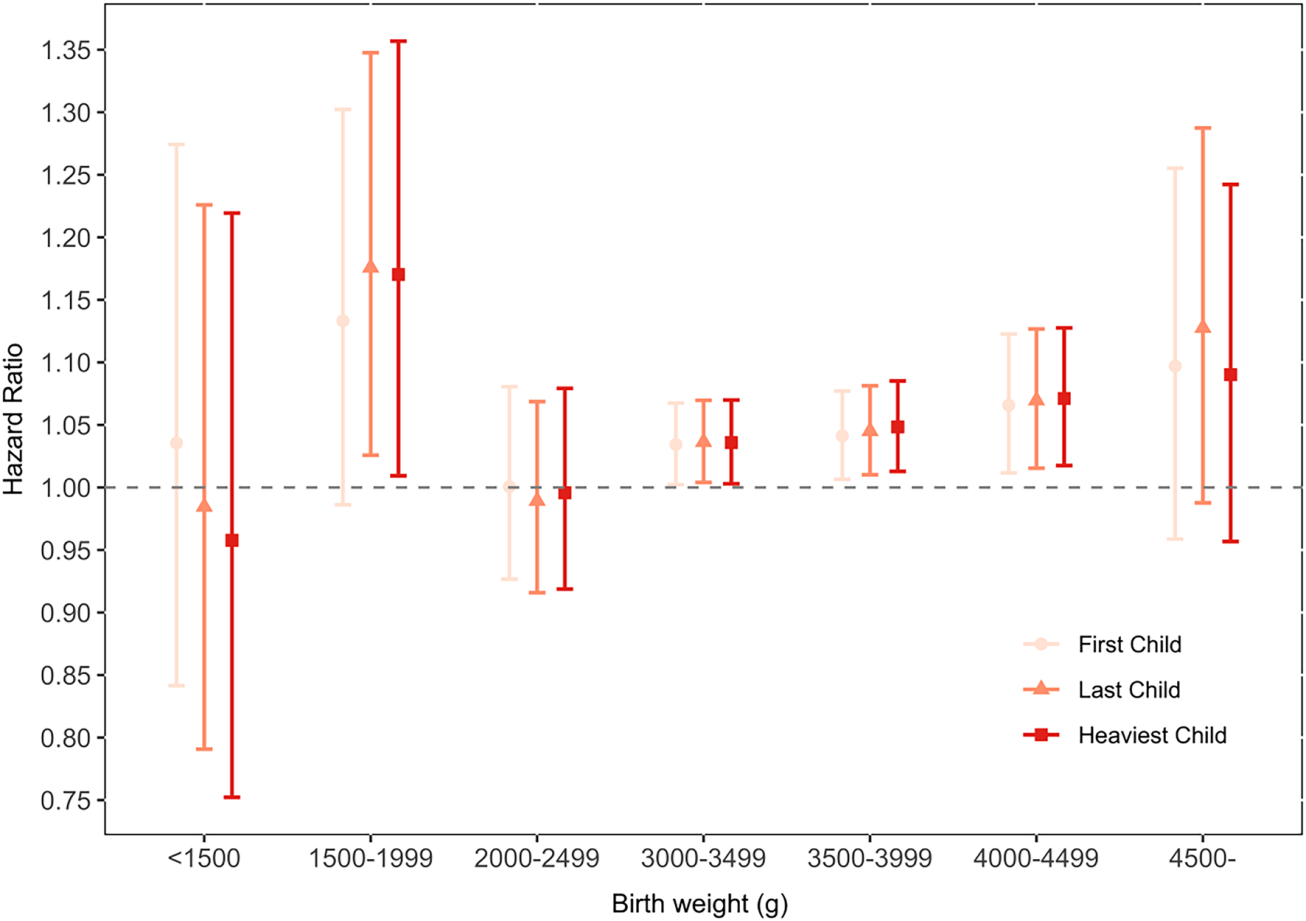
Long-term hazard ratio of cancer after childbirth, according to fetal birth weight. *Birth weight between 2500 and 2999 was control group.

Although the association between fetal macrosomia and subsequent maternal cancer was not significant in this study, we divided the birth weight into groups of 500 g incremental increases to further analyze the relationship between birth weight subgroups and maternal cancer (Fig. 4). We found that groups with birth weights of more than 2500 g were associated with a substantially increased risk of maternal cancer, with the risk increasing as birth weight increased (on average 1.9%, 95% CI 0.5–3.3%, risk increase per 500 g, data not shown). Moreover, we noted that with the last and heaviest deliveries, the risk of maternal cancer was significantly increased in the group with birth weights of 1500–1999 g (HR = 1.18, 95% CI 1.03–1.35 and HR = 1.17, 95% CI 1.01–1.36, respectively).

## Discussion

Maternal hormone incretion levels change dramatically during pregnancy, which influence fetal growth through growth factor pathways^14–16^. For instance, estrogen^17,18^ and IGF-1 in mid-pregnancy^19^ stimulate fetal growth, which may affect birth weight and induce LGA infants. Estrogen and IGF-1 receptors have also been shown to regulate cell proliferation and resistance to apoptosis^20,21^. Moreover, high estrogen and IGF-1 levels in adulthood have been associated with the risk of many types of cancer^22,23^. Additionally, serum leptin concentrations increase during pregnancy, particularly in women who gain an excessive amount of weight^24^. These women are also more likely to develop breast cancer after menopause compared with women who do not exceed pregnancy weight recommendations^25^. Based on this underlying biological mechanism, in this large population-based cohort study, we focused on the incidence of cancer in mothers after childbirth and the association between fetal growth and maternal malignancy. This study found that EFG was associated with an increased risk of maternal cancer after controlling for maternal demographic and pregnancy characteristics, such as year of childbirth, maternal age at birth, maternal education level, number of childbirths, history of abortion, number of embryos, caesarian, child gender, and gestational age.

The most substantial finding of our study was the effect of birth weight and LGA birth on the risk of maternal malignancy. In this study, we found that LGA births were associated with an increased risk of subsequent maternal cancer. That is, women who gave birth to LGA infants had a higher risk of developing subsequent malignancies. According to former international studies, small for gestational age (SGA) was reported as a protective factor for maternal breast and ovarian cancer, and high birth weight increased the risk of maternal cancer^6,26^. Although EFG is associated with an increased risk of developing hormone-related cancer in mothers^4,7^, the evidence for the association is still limited and requires further research, as previous studies have not included Asian populations. Our study further confirmed the association between LGA births and the increased risk of subsequent maternal cancer, extending the literature to Asian mothers. However, in this study, we did not note the association between fetal macrosomia and an increased risk of maternal cancer, which has been reported in previous studies^27–30^. The reason that the association between fetal macrosomia and subsequent maternal cancer was not significant in our findings may be that premature and low birth weight were also risk for maternal cancer^26,31^. Simple classifications into macrosomia and non-macrosomia groups may not show the full impact of birth weight as both low and high birth weights were risk factors for subsequent maternal cancer. To study the impact of different birth weight groups with more detail, we divided birth weight into subgroups of 500 g increments. The significant association between birth weight and the risk of maternal cancer became more apparent after the birth weight division, as we noted a substantial increase in the risk of maternal cancer associated with an increase in birth weight over 2500 g. Moreover, this study was the first to attempt to find a more appropriate birth weight to reduce future maternal cancer risk, as our findings showed that it is better to control the birth weight around 2500 g to lower the risk of subsequent maternal cancer.

Additionally, our study confirmed that low birth weight, which was strongly associated with premature births, was also associated with subsequent maternal cancer, as we unexpectedly observed that the risk of maternal cancer was highest in the group with birth weights of 1500–1999 g. Although this finding requires verification, it suggests that controlling birth weight and avoiding premature birth is crucial to reducing the risk of subsequent maternal cancer. This may be achieved by determining the factors of preterm births and low birth weights, including eclampsia and gestational diabetes mellitus.

Most former studies have always included only macrosomia as an indicator of fetal growth level. Macrosomia, usually defined as infant birth weight of ≥ 4000 g, does not consider gestational age, gender, or region-specific differences in mean birth weight and maternal body weight. One study among Asian population^32^ showed that reporting LGA, using standardized regional growth charts, would better capture the incidence of high birth weight and allow for the comparison and identification of contributing factors. From 2016 to 2017, the prevalence of macrosomia in China was 7.37%, while the prevalence of LGA was double, at 15.5%^19^. Our study added LGA as another indicator to show the extent of fetal growth. Compared with macrosomia, LGA may be a more comprehensive and sensitive index reflecting fetal growth, by considering gestational age and gender factors. LGA can reflect EFG in each period of gestation, instead of macrosomia, which may only reflect the infant's weight at birth. For example, a preterm birth who has overgrown at early gestation can be an LGA birth, but may not indicate fetal macrosomia, with normal birth weight. Consequently, it is better for us to analyze both indices—macrosomia and LGA. The association between LGA and maternal cancer was statistically significant, indicating that the gestational age-specific weight along with the duration of the pregnancy is crucial, instead of controlling the birth weight within a specific span only.

Apart from the fetal growth results, our study also found that women who gave birth at a relatively later age were more likely to develop subsequent cancers; this outcome was confirmed by further analysis which supported this finding^33^. Moreover, delivery before the age of 24 years decreased long-term cancer risk, while delivery after the age of 26 years increased long-term cancer risk, indicating that conceiving earlier may reduce the risk of subsequent cancer.

Our findings from a large, population-based cohort contribute to the existing evidence regarding the role of EFG on subsequent malignancy risk in the mother. Previous studies appeared to focus on hormone-related cancers, except one large cohort study in Sweden, which found that EFG was independently associated with a higher risk of colorectal cancer. Our study included all types of maternal cancers. In addition, we included the variable LGA, a more accurate and proper index, to study the association between fetal growth and maternal cancer. Furthermore, no study has been able to find a more appropriate birth weight to reduce the future maternal cancer risk, whereas we determined the optimal birth weight to be approximately 2500 g for the lowest risk of all maternal cancers.

Limitations included a lack of information for certain other exposures that may be associated with maternal malignancies, including alcohol use, smoking, and diet. Hence, we were unable to examine their potential influences on our findings. This cohort was also relatively young, with a median age of 37 years (maximum 65 years) at the end of follow-up. Additional follow-up is required to examine our observed associations at older ages, when malignancy is more common. Studies with more specific types of cancer would be useful to examine the associations reported in this study. Although the cases in our model were independent, as we divided birth records into three groups as “the first group, the last group and the heaviest group”, the births from the same women were not studied separately, and this should be analyzed in the future.

In conclusion, the association between LGA birth and increased risk of maternal malignancy was statistically significant and plausible in our study. This potential link should be further examined in a different population. The results of this study may provide a basis for future research on the link between fetal growth and the risk of maternal malignancy.

## Data Availability

Our data resources were related to three data register systems, so the data that support the findings of this study cannot be shared without the consensus of the three departments. The data that support the findings of this study can be accessed by contacting the corresponding author, upon reasonable request.

## References

[1] Markestad T, Bergsjø P, Aakvaag A (1997). Prediction of fetal growth based on maternal serum concentrations of human chorionic gonadotropin, human placental lactogen and estriol. Acta Obstet. Gynecol. Scand. Suppl..

[2] Gardner M, Goldenberg RL, Cliver SP (1995). Maternal serum concentrations of human placental lactogen, estradiol and pregnancy specific beta 1-glycoprotein and fetal growth retardation. Acta Obstet. Gynecol. Scand. Suppl..

[3] Troisi R, Bjørge T, Gissler M (2018). The role of pregnancy, perinatal factors and hormones in maternal cancer risk: A review of the evidence. J. Intern. Med..

[4] Swerdlow AJ, Wright LB, Schoemaker MJ, Jones ME (2018). Maternal breast cancer risk in relation to birthweight and gestation of her offspring. Breast Cancer Res..

[5] Crump C, Sundquist J, Sieh W (2016). Fetal growth and subsequent maternal risk of thyroid cancer. Int. J. Cancer.

[6] Mucci LA, Dickman PW, Lambe M (2007). Gestational age and fetal growth in relation to maternal ovarian cancer risk in a Swedish cohort. Cancer Epidemiol. Biomark. Prev..

[7] Crump C (2015). Fetal growth and subsequent maternal risk of colorectal cancer. Cancer Epidemiol. Biomark. Prev..

[8] Ghosh RE, Berild JD, Sterrantino AF (2018). Birth weight trends in England and Wales (1986–2012): Babies are getting heavier. Arch. Dis. Child. Fetal Neonatal Ed..

[9] Lu Y, Zhang J, Lu X (2011). Secular trends of macrosomia in southeast China, 1994–2005. BMC Public Health.

[10] Sammel MD (2018). Big data approach to evaluation of birth defects and assisted reproductive technology: The Chinese linkage cohort. Fertil. Steril..

[11] Yu HT, Yang Q, Sun XX (2018). Association of birth defects with the mode of assisted reproductive technology in a Chinese data-linkage cohort. Fertil. Steril..

[12] Bao PP, Zheng Y, Wang CF, Gu K, Jin F, Lu W (2009). Time trends and characteristics of childhood cancer among children age 0–14 in Shanghai. Pediatr. Blood Cancer.

[13] Sjaarda LA, Albert PS, Mumford SL, Hinkle SN, Mendola P, Laughon SK (2014). Customized large-for-gestational-age birthweight at term and the association with adverse perinatal outcomes. Am. J. Obstet. Gynecol..

[14] Troisi R, Potischman N, Roberts J (2003). Associations of maternal and umbilical cord hormone concentrations with maternal, gestational and neonatal factors (United States). Cancer Causes Control.

[15] David PJ, Hulka BS, Savitz DA (2003). Accuracy of fetal growth indicators as surrogate measures of steroid hormone levels during pregnancy. Am. J. Epidemiol..

[16] Nagata C, Iwasa S, Shiraki M (2006). Estrogen and alpha-fetoprotein levels in maternal and umbilical cord blood samples in relation to birth weight. Cancer Epidemiol. Biomark. Prev..

[17] Kaijser M, Granath F, Jacobsen G (2000). Maternal pregnancy estriol levels in relation to anamnestic and fetal anthropometric data. Epidemiology.

[18] Lagiou P, Samoli E, Hsieh CC (2014). Maternal and cord blood hormones in relation to birth size. Eur. J. Epidemiol..

[19] Iii J, Schmidt S, Paranjape A (2004). Maternal insulin-like growth factor-I levels (IGF-I) reflect placental mass and neonatal fat mass. Am. J. Obst. Gynecol..

[20] Bartella V, De Marco PD, Malaguarnera R, Belfiore A, Maggiolini M (2012). New advances on the functional cross-talk between insulin-like growth factor-I and estrogen signaling in cancer. Cell Signal.

[21] Pollak M, Schernhammer ES, Hankinson SE (2008). Insulin, Insulin-like growth factors and neoplasia. Best Pract. Res. Clin. Endocrinol. Metab..

[22] Key TJ, Appleby PN, Reeves GK (2010). Insulin-like growth factor 1 (IGF1), IGF binding protein 3 (IGFBP3), and breast cancer risk: Pooled individual data analysis of 17 prospective studies. Lancet Oncol..

[23] Wei EK, Ma J, Pollak MN (2005). A prospective study of C-peptide, insulin-like growth factor-I, insulin-like growth factor binding Protein-1, and the risk of colorectal cancer in women. Cancer Epidemiol. Biomark. Prev..

[24] Hardie L, Trayhurn P, Abramovich D, Fowler P (1997). Circulating leptin in women: A longitudinal study in the menstrual cycle and during pregnancy. Clin. Endocrinol. (Oxf.).

[25] Kinnunen TI, Pasanen M, Aittasalo M (2007). Preventing excessive weight gain during pregnancy—A controlled trial in primary health care. Eur. J. Clin. Nutr..

[26] Nechuta S, Paneth N, Pathak DR, Gardiner J, Copeland G, Velie EM (2010). A population-based case-control study of fetal growth, gestational age, and maternal breast cancer. Am. J. Epidemiol..

[27] Ahlgren M, Wohlfahrt J, Olsen LW (2010). Birth weight and risk of cancer. Cancer.

[28] Mccormack VA, Silva I, Koupil I (2005). Birth characteristics and adult cancer incidence: Swedish cohort of over 11,000 men and women. Int. J. Cancer.

[29] Crump C, Sundquist K, Sieh W, Winkleby MA, Sundquist J (2012). Perinatal and family risk factors for Hodgkin lymphoma in childhood through young adulthood. Am. J. Epidemiol..

[30] Crump C, Sundquist K, Sieh W, Winkleby MA, Sundquist J (2012). Perinatal and family risk factors for non-Hodgkin lymphoma in early life: A Swedish national cohort study. J. Natl. Cancer Inst..

[31] Ardalan A, Bungum T (2016). Gestational age and the risk of maternal breast cancer: A population-based case–control study. Breast J..

[32] Harvey L, van Elburg R, van der Beek EM (2021). Macrosomia and large for gestational age in Asia: One size does not fit all. J. Obstet. Gynaecol. Res..

[33] Guihua L, Li L, Wenxian L (2020). Pregnancy complicated with severe special diseases in 2014–2016 in Shanghai: Its risk management study. Shanghai J. Prev. Med..

